# A new approach to RNA synthesis: immobilization of stably and functionally co-tethered promoter DNA and T7 RNA polymerase

**DOI:** 10.1093/nar/gkae599

**Published:** 2024-07-16

**Authors:** Kithmie MalagodaPathiranage, Ruptanu Banerjee, Craig T Martin

**Affiliations:** Department of Chemistry, University of Massachusetts Amherst, Amherst, MA 01003, USA; Department of Chemistry, University of Massachusetts Amherst, Amherst, MA 01003, USA; Department of Chemistry, University of Massachusetts Amherst, Amherst, MA 01003, USA

## Abstract

Current approaches to RNA synthesis/manufacturing require substantial (and incomplete) purification post-synthesis. We have previously demonstrated the synthesis of RNA from a complex in which T7 RNA polymerase is tethered to promoter DNA. In the current work, we extend this approach to demonstrate an extremely stable system of functional co-tethered complex to a solid support. Using the system attached to magnetic beads, we carry out more than 20 rounds of synthesis using the initial polymerase-DNA construct. We further demonstrate the wide utility of this system in the synthesis of short RNA, a CRISPR guide RNA, and a protein-coding mRNA. In all cases, the generation of self-templated double stranded RNA (dsRNA) impurities are greatly reduced, by both the tethering itself and by the salt-tolerance that local co-tethering provides. Transfection of the mRNA into HEK293T cells shows a correlation between added salt in the transcription reaction (which inhibits RNA rebinding that generates RNA-templated extensions) and significantly increased expression and reduced innate immune stimulation by the mRNA reaction product. These results point in the direction of streamlined processes for synthesis/manufacturing of high-quality RNA of any length, and at greatly reduced costs.

## Graphical Abstract

**Graphical Abstract:**
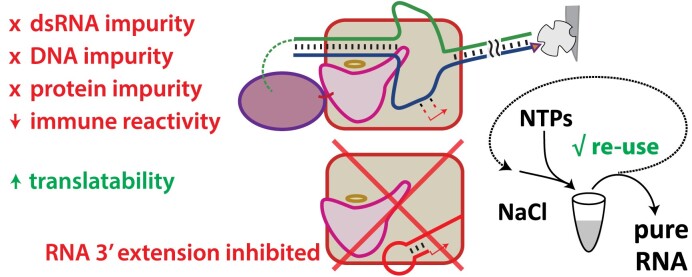

## Introduction

During traditional batch synthesis of RNA using T7 RNA polymerase, longer than encoded by-products are often observed (1). These by-products can arise from multiple different pathways, including *cis*-primed (hairpin-initiated) extension, *trans-*primed extension and 3′ overhang initiation (1–5). We recently carried out an in-depth sequence analysis on these longer RNAs and showed that the majority of longer RNA arise from encoded full length (runoff) RNA looping back on itself to form short polymerase-bound hairpins that can serve as primers for *cis*-templated elongation, a phenomenon called *cis*-primed extension. These *cis*-primed extensions are driven by the higher yield conditions used in traditional batch RNA synthesis, in which the concentration of the desired runoff RNA increases as the reaction proceeds, which in turn drives *cis*-primed extension to compete more effectively with (desired) promoter rebinding (2,5,6).

Since the RNA extensions are self-templated, the product is (partially) double stranded RNA (dsRNA). For RNA therapeutics, it is essential to remove these double stranded impurities since double stranded regions longer than about 40 bp can stimulate the innate immune response, as cells recognize them as a sign of viral attack (7–10).

In the process of synthesizing RNA, several complex steps of purification are required, involving chromatographic and/or affinity techniques (11–13). These purification steps are used to reduce the concentrations of longer RNA products that can arise during transcription. Among the undesired products are very short RNA by-products, such as 2–8 bp abortive RNAs, which can be easily eliminated from the transcription reaction post-synthesis.

However, the real challenge lies in purifying the longer RNA impurities, which prove to be more stubborn to remove while trying to retain the correct length RNA. The primary difficulty arises due to the presence of polydispersity in RNA lengths, making their selective isolation arduous. In response to this challenge, some researchers have explored the use of cellulose-modified membranes, which have shown promise in selectively removing dsRNA molecules (11). Although these membranes exhibit considerable effectiveness, achieving a 100% separation remains difficult. For RNAs with an encoded polyA tail, affinity purification with dT25 is increasingly common, but with polyA tails of 60–120 bp, RNAs with extensive double stranded region(s) can be inadvertently captured (14). Despite the purification efforts, this multi-step processing is often unable to eliminate the impurities, leading to lower yields of the desired final RNA product. This decrease in yield is a significant concern, especially in clinical trials where precise and consistent RNA products are essential. Residual dsRNA can cause serious complications in clinical applications, potentially leading to failures in clinical trials (15).

Currently there are few methods published to decrease the amount of double stranded by-products formed during transcription (16–20). We have recently demonstrated that favoring promoter binding by increasing the affinity between (modified) promoter DNA and wild type T7 RNA polymerase, allows the use of elevated salt conditions (17). The corresponding increase in ionic strength weakens RNA rebinding to the enzyme to limit *cis*-primed extension. Similarly, tethering StrepTag-labeled T7 RNA polymerase to biotin tagged promoter DNA via the intermediate surface of (tetrameric) Strep-Tactin®XT strengthens binding due to the increased local concentration of the promoter DNA relative to its promoter recognition site on the protein (21). This effective strengthening of promoter binding renders salt-resistant transcription and increasing salt leads to a decrease in cis-primed dsRNA by-products. An additional benefit of this approach, with both enzyme and DNA tethered to magnetic beads, is that it is reusable. However, since biotin binding to Strep-Tactin®XT is not as strong as its binding to native streptavidin (22,23), some activity is lost with each wash, limiting reusability.

In the current work, we present a significantly improved method of increasing the local concentration of promoter DNA near the promoter binding site, through the covalent tethering of T7 promoter DNA near the promoter binding site of the RNA polymerase. In this approach, we fuse the HaloTag domain (24) to the N-terminus of T7 RNA polymerase and then react that species with promoter DNA containing a linker-chloroalkane functionality, to achieve a fully covalent connection between the enzyme and the promoter DNA. As predicted, the tethered system allows transcription with increased salt tolerance. Tethering this system to magnetic beads additionally allows for a reusable system. We demonstrate that this new system can undergo many rounds of transcriptions to yield very large quantities of runoff RNA. Notably, the resultant mRNA exhibits exceptional translatability in mammalian cells, coupled with minimal inadvertent activation of innate immune responses. Our methodology represents a pioneering advancement in mRNA manufacturing by *in vitro* transcription (IVT), as it achieves the dual objective of low immunogenicity and high translational efficiency without recourse to modified bases or extensive purification procedures. This study stands as one of the earliest documented instances of such achievement within the scientific literature (17,25).

## Materials and methods

### Oligonucleotides

Oligonucleotides were obtained from Integrated DNA Technologies (IDT). Template strands and downstream primers were purchased with the 5′ biotin modification where indicated. Nontemplate strands and upstream primers were purchased with 5′ amino modifier C6 or C12, with or without internal spacers, as indicated. Syntheses of short RNA contained an amino modifier C6, while PCR primers for nanoluciferase (NLuc) mRNA expression contained an amino modifier C12 followed by four sequential spacer 18 monomers (both variations function well).

**Figure 1.**
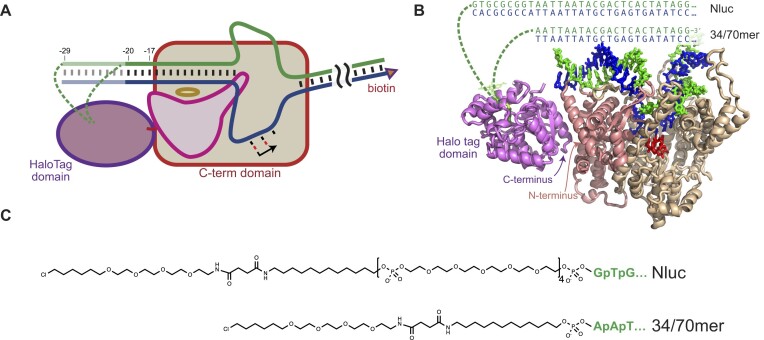
HaloTag Domain fused to T7 RNA polymerase. (**A**) Simplified representation. The N-terminal domain (pink) together with the specificity loop (dark yellow oval) comprise the promoter binding element. Green dashed lines indicate the linker connecting DNA to the HaloTag active site. (**B**) Juxtaposition of the crystal structure of promoter bound T7 RNA polymerase (1QLN, with downstream DNA modeled from 1MSW) and the HaloTag domain (7ZIY). In this portrayal, the HaloTag domain is placed with its C-terminus near the N-terminus of T7 RNA polymerase and is rotated to place its active site opposite the projection of the upstream DNA. (**C**) Linkers used to connect the alkyl halide HaloTag ligand to the DNA.

Where present, the primary amine was then coupled at room temperature overnight with a 1:20 ratio of DNA: HaloTag^TM^ succinimidyl ester (O4) ligand (Promega) in PBS buffer. The final structures are shown in Figure 1 and the reactions are described in Supplementary Figure S1. The modified DNA was purified via reverse phase high performance chromatography (HPLC Agilent 1100).

T7 RNA polymerase containing an N-terminal His-tag was prepared by expression in *Escherichia coli* strain BL21 carrying the plasmid pBH161, containing the T7 RNA polymerase gene under control of the LacZ promoter (26).

To prepare the HaloTag-fused RNA polymerase construct, DNA encoding the HaloTag protein sequence was PCR amplified from pDB-His6-MBP-Halo-NZF1, a gift from Eric Streiter (27) who developed the plasmid for expression in *E. coli* cells from an earlier reported plasmid that was used to express HaloTag fused proteins in mammalian cells (24). The HaloTag domain was inserted in-frame immediately upstream of the N-terminal domain of T7 RNA polymerase in the pBH161 plasmid. As this new construct retains a His-tag element, purification followed that of wild type T7 RNA polymerase.

### Co-immobilization of short DNA and HaloTag T7 RNA polymerase on beads

To achieve a covalent crosslink, the above chloro-alkyl nontemplate DNA strand was first incubated with the HaloTag-fused RNA polymerase at 37°C for 2 h in a 1:2 enzyme: DNA ratio in PBS (phosphate buffered saline). For untethered experiments, an amount of the appropriate template strand equal to that of the nontemplate strand was then added to complete the complex. As indicated below the nontemplate strand was either fully complementary to the template strand or was truncated near the promoter (both constructs are generally functional in transcription). The sequence of nontemplate and template strands for short DNA are depicted in Supplementary Table S1.

To bind the complex to magnetic beads the complementary template strand of the DNA containing a Biotin-TEG (triethyleneglycol) at its 5′ (downstream) end was incubated with 400 pmol/mg streptavidin magnetic beads (New England Biolabs) at room temperature for 0.5 h. The complex was then washed with washing buffer (0.5 M NaCl, 20 mM Tris–HCl (pH 7.5), 1 mM ethylenediaminetetraacetic acid (EDTA)) three times.

The bead-bound template strand was then incubated with the above protein-tethered nontemplate strand complex for 0.5 h in the same washing buffer, to allow annealing of the complementary strands. The complex was then washed three times and resuspended in a volume of 1X transcription buffer (40 mM magnesium acetate, 30 mM 4-(2-hydroxyethyl)-1-piperazineethanesulfonic acid (HEPES), 25 mM potassium glutamate, 0.25 mM EDTA and 0.05% Tween-20) to generate a concentrated ≈10 μM stock bead solution for downstream transcription reactions. Note that the washing steps should remove free enzyme and uncomplexed nontemplate DNA.

### *In vitro* transcription reactions for short RNA

Untethered transcription reactions contained 1 μM of the indicated (partially duplex) DNA and 1 μM native or HaloTag-coupled T7 RNA polymerase. Bead-immobilized reactions contained approximately 0.5 μM complex.

The transcription mix contained 7.5 mM each of guanosine triphosphate (GTP), cytidine triphosphate (CTP), adenosine triphosphate (ATP) and uridine triphosphate (UTP), 40 mM magnesium acetate, 30 mM HEPES, 25 mM potassium glutamate, 0.25 mM EDTA and 0.05% Tween-20 and were supplemented with 2000 U/ml of RNase Inhibitor, Murine (New England Biolabs) and 5 U/ml of inorganic pyrophosphatase (yeast, New England Biolabs). NaCl was included as indicated. Reactions were carried out at 37°C for 4 h. RNA was purified with the Monarch® RNA Cleanup Kit (New England Biolabs) to remove NTPs and enzymes.

### Repeat batch (tethered) synthesis of short RNA

Reactions were carried out at 37°C for 1.5 h in a transcription mix with 5 mM each NTPs supplemented with 300 mM NaCl. To keep beads suspended, the tubes were place on a rotor, turning at approximately 40 revolutions per min. After each transcription reaction, the immobilized enzyme–DNA complex was separated from the reaction mix via a magnet, and then washed once with washing buffer. Fresh transcription mix was then added to initiate a new reaction. This was repeated 30 times over 10 days, and between days, the beads were stored at 4°C. RNA was purified with the Monarch® RNA Cleanup Kit (New England Biolabs) to remove NTPs and enzymes.

### Nanoluciferase mRNA template design

NLuc plasmid NanoLuc-SZ2, was a gift from Daniel Hammer: Addgene plasmid #176645; (http://n2t.net/addgene:176645; RRID: Addgene_176645) (28). The nanoluciferase-encoding gene (Supplementary Table S2, 1 and 2), to be used for the experiments was amplified using primers (Supplementary Table S2, 3).

### Co-immobilization of long DNA and HaloTag T7 RNA polymerase on beads

The NLuc mRNA template was PCR amplified with a chloro-alkyl modified forward primer and a biotin-modified reverse primer. After purification the complex was incubated with HaloTag T7 RNA polymerase 37°C for 2 h in a 1:2 enzyme:DNA ratio.

The assembled enzyme-DNA complex was incubated with 50 pmol/mg streptavidin magnetic beads (New England Biolabs) at room temperature for 0.5 h. The complex was then washed with washing buffer three times. As noted above, this washing should remove all unbound RNA polymerase, as illustrated in supplemental Figure S3.

### *In vitro* transcription reaction for long RNA

High yield (untethered) transcription reactions contained 0.3 μM of the indicated (fully duplex) DNA and 0.15 μM native or HaloTag-coupled T7 RNA polymerase. Bead-bound complex reactions contained approximately 0.15 μM complex.

The transcription mix contained 7.5 mM each of GTP, CTP, ATP and UTP, 40 mM magnesium acetate, 30 mM HEPES, 25 mM potassium glutamate, 0.25 mM EDTA and 0.05% Tween-20 and were supplemented with 2000 U/ml of RNase Inhibitor, Murine (New England Biolabs) and 5 U/ml of inorganic pyrophosphatase (yeast, New England Biolabs). NaCl was added as indicated. Reactions were carried out at 37°C for 2 h. RNA was purified with the Monarch® RNA Cleanup Kit (New England Biolabs) to remove NTPs and enzymes.

### Repeat batch (tethered) synthesis of long RNA

Reactions were carried out at 37°C for 2 h in the above transcription mix supplemented with 300 mM NaCl. To keep beads suspended, the tubes were placed on a rotor, turning at approximately 40 revolutions per min. After each transcription reaction, the immobilized enzyme-DNA complex was separated from the reaction mix via a magnet, and then washed once with washing buffer. Fresh transcription mix was then added to initiate a new reaction. This was repeated multiple times. RNA was purified with the Monarch® RNA Cleanup Kit (New England Biolabs) to remove NTPs and enzymes.

### IVT Kit

HiScribe® T7 High Yield RNA synthesis Kit was used for carrying out IVT of 70mer RNA and NLuc mRNA with 0.5 μM and 0.15 μM of respective DNAs. The transcription mix contained reaction buffer (provided in the kit) and supplemented with 7.5 mM each of GTP, CTP, ATP and UTP. Reactions were carried out at 37°C for 2 h and RNA was purified with the Monarch® RNA Cleanup Kit (New England Biolabs) to remove NTPs and enzymes.

### mRNA Capping

*In vitro* transcribed mRNA after column purification was capped using mRNA cap 2′-*O*-methyltransferase (New England Biolabs) and Vaccinia Capping Enzyme (New England Biolabs) according to manufacturer's recommendation. The mRNA, after capping, was used directly for transfecting cells without any other purification.

### NLuc mRNA transfection and translation assay

HEK293T cells were maintained in Dulbecco's modified Eagle's medium (DMEM), high glucose (Gibco™-11965118) with 10% fetal bovine serum (FBS) and 1% penicillin–streptomycin (Gibco™). The cells were seeded 24 h prior to the experiment in a 96-well plate (Nunc™ Edge™, ThermoFisher Scientific) at a concentration of 10^5^ cells/ml in a total volume of 100 μl/well. The cells were transfected with NLuc mRNA using Lipofectamine™ MessengerMax™ (Invitrogen) as per manufacturer's protocol. Briefly, 100 ng of NLuc mRNA was mixed with 0.3 μl of Lipofectamine™ MessengerMax™ and incubated at 25°C for 5 min. The mRNA-lipid complex was then added directly to the growing media of HEK293T cells. NLuc mRNA translation in HEK293T cells were evaluated after 48 h using Nano-Glo® Luciferase Assay System (Promega) as per manufacturer's recommendation. Briefly, 100 μl of Nano-Glo® Luciferase Assay Substrate (diluted 1:50 with Nano-Glo® Luciferase Assay Buffer) was added to each well containing 100 μl media. The luminescence was recorded after 5 min using SpectraMax M5 (Molecular Devices). A background luminescence was measured with HEK293T cells, treated as above, but without any mRNA transfection.

### Innate immune response analysis using RT-qPCR

Cellular mRNA was harvested 48 h post-transfection using Luna® Cell Ready Lysis Module (New England Biolabs). From 2 μl of lysate, Luna Universal One-Step RT-qPCR Kit was used to generate cDNA of the genes of interest and quantify those cDNAs in a single step, as per manufacturer's protocol. The sequences of the primers used for the RT-qPCR are in Supplementary Table S3. Cells treated with high molecular weight (HMW) polyinosinic-polycytidylic acid (poly (I:C)) (Invivogen) was used as positive control (extracellular dsRNA mimic) and DPBS was used as negative control for RT-qPCR analysis.

The relative fold change was calculated using the ($2 - {\mathrm{\Delta \Delta Ct}}$) delta-delta C_t_ method (29). Glyceraldehyde-3-phosphate dehydrogenase (GAPDH) was used as the housekeeping gene.

## Results

T7 RNA polymerase is widely used in the *in vitro* synthesis of RNA. However, high yield transcription is plagued by the unintended production of dsRNA by-products that must be removed post-synthesis through purifications like HPLC, gel electrophoretic, or cellulose base chromatography (11,12). The aim of this work is to eliminate the formation of dsRNA during transcription (1,2,4,5).

As noted above, functionally coupling T7 RNA polymerase to its promoter DNA brings the two in close proximity, allowing salt-resistant initiation (21,30). This allows higher RNA yields at elevated salt conditions that reduce *cis-*primed extension products (21). Tethering this system to magnetic beads allows it to be reused multiple times, but since in our recent work, the tethering is not covalent and the Strep-Tactin®XT mutation weakens biotin binding, transcription yields decrease with each cycle of washing.

To overcome the loss of activity with washing, in the current work we develop a covalent coupling between protein and DNA by genetically fusing the HaloTag domain, in frame, to the amino terminus of T7 RNA polymerase and then coupling modified DNA to the HaloTag domain. The HaloTag domain is widely used for covalently attaching molecules to proteins (24), and protein domains have been genetically fused to the N-terminus of T7 RNA polymerase while retaining full polymerase function (31). Fusion of the HaloTag domain to the N-terminus of RNA polymerase should place molecules bound to the HaloTag domain in the vicinity of the DNA upstream end of the promoter, as loosely illustrated schematically in Figure 1A. The manually docked representation in Figure 1B shows a HaloTag domain placement that localizes its C-terminus near the N-terminus of T7 RNA polymerase (and further, is in a rotational state that places the HaloTag active site towards the upstream end of promoter DNA bound to T7 RNA polymerase).

To demonstrate functionality, we conducted an initial experiment involving transcription of a 34 bp RNA from synthetic promoter DNA containing the short linkage (34/70mer) shown in Figure 1C (its construction is detailed in Supplementary Figure S1). Our primary objective was to assess whether these modifications allowed promoter binding, initiation, and promoter escape. The gel analysis in Supplementary Figure S2 shows similar transcription profiles for the crosslinked species and for the non-crosslinked controls. These data indicate that both the HaloTag fusion to the N-terminus of T7 RNA polymerase and the covalent labeling process have no adverse effects on the enzyme's inherent functionality.

### Tethered transcription at high salt decreases cis-primed extension

**Figure 2.**
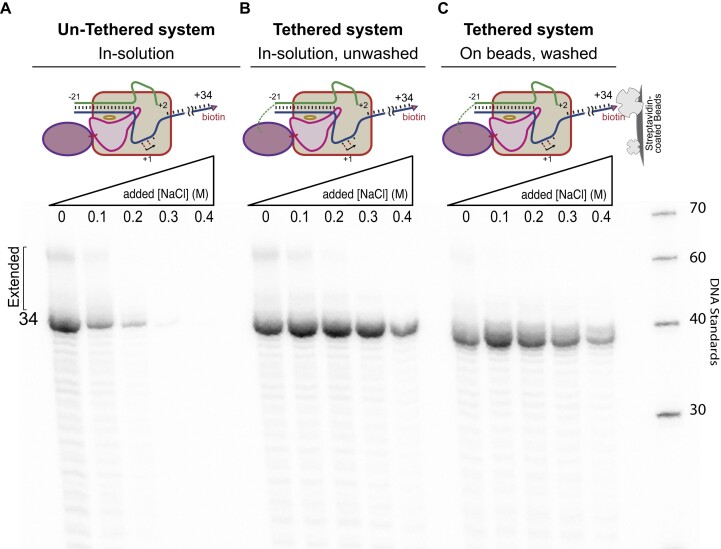
Assessing salt tolerance of untethered and tethered constructs. Salt dependence profiles of (**A**) untethered (free in solution) promoter and enzyme, (**B**) co-tethered complex (in solution) and (**C**) co-tethered complex bound to Streptavidin-coated magnetic beads and washed 3× in 0.5 M NaCl buffer before transcription.

Transcription initiation, but not elongation, is sensitive to high salt concentrations (30,32). Similarly, self-primed extension to generate dsRNA is also sensitive to elevated salt (17,21). Initiation and self-primed extension each require binding of a nucleic acid to the protein. Tethering of promoter DNA close to the T7 promoter recognition site has been shown to strengthen promoter specific transcription by establishing a high local (relative) concentration of the two binding partners, allowing resistance to salt (21,30). To confirm this expectation, we incubated HaloTag T7 RNA polymerase with a promoter nontemplate DNA strand (spanning positions −20 to +2), with and without a chloroalkyl modification at the 5′ end. We then added the template strand encoding a 34mer RNA known to carry out self-primed extension (2) and transcribed under increasing salt conditions. The data presented in Figure 2A show that the uncrosslinked control is salt sensitive at 0.1 M added NaCl, as before (17,21,32).

In contrast, the results in Figure 2B show that the crosslinked construct shows good salt resistance up to at least 0.3 M added NaCl. These results confirm our previous observation that tethering the promoter close to the promoter binding site of T7 RNA polymerase drives increased promoter specific binding by T7 RNA polymerase (21).

Incomplete coupling of HaloTag-RNA polymerase to its promoter DNA would leave residual unbound free enzyme in solution, which in turn could bind released RNA and extend it to form 3′-prime extended products. Such uncoupled species would also be salt-sensitive (in both promoter binding and RNA re-binding). To fully remove the uncoupled enzyme from solution, we attached the co-tethered system to Streptavidin-coated magnetic beads via a biotin functionalization at the downstream 5′ end of the DNA. This allows washing of the complex in 0.5 M added NaCl buffer to remove free enzyme (and DNA), as described and demonstrated in Supplementary Figure S3. Transcription from washed, co-immobilized complexes is presented in Figure 2C, and the results show a decrease in 3′-prime extended products, even at low salt concentrations.

The encoded RNA sequence used in these experiments is recognized for its tendency towards cis-primed extension (17), as observed in the low salt lanes of Figure 2A and B. As observed previously, the fraction of RNA that is converted into cis-primed dsRNA decreases with increasing salt, even when the overall yield of RNA remains approximately constant. Note that even at low salt, the fully washed co-complex shows reduced *cis*-primed extensions, as seen in Figure 2C. This is consistent with tethering of the polymerase near its promoter binding site providing a competitive advantage over RNA re-binding to the polymerase.

### Tethering restricts transcription to the bound promoter

**Figure 3.**
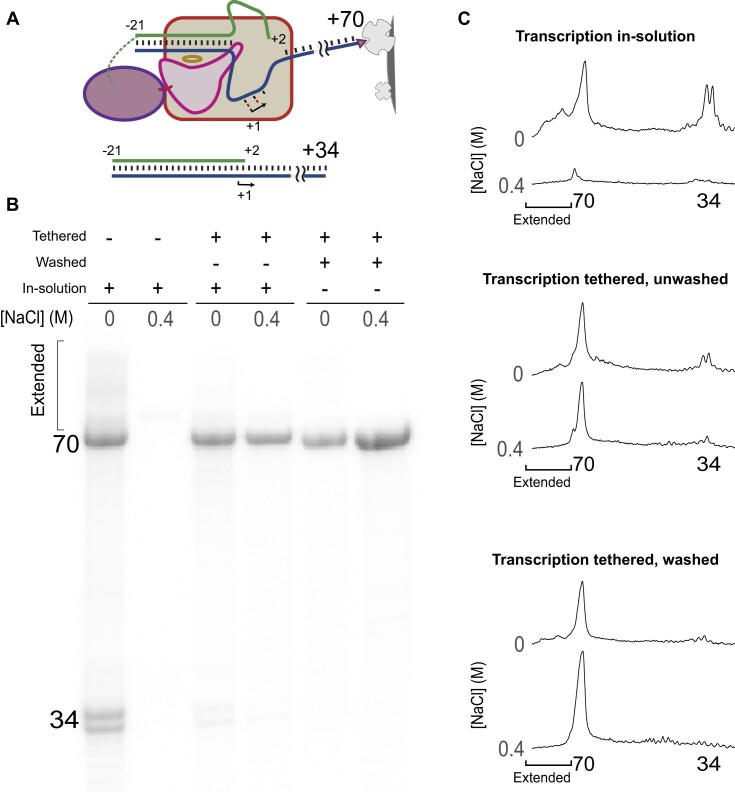
Tethering drives transcription to the crosslinked DNA partner. (**A**) Test system encodes a 70mer RNA and in all cases is co-incubated with free DNA encoding a 34mer RNA. (**B**) RNA products produced under three conditions (see text), each at 0 and 0.4 M added NaCl. (**C**) Individual lane tracings quantify expression from 70mer-encoding DNA and from the challenge 34mer encoding DNA.

To further demonstrate that tethering the promoter DNA to the enzyme advantages (nearby) promoter-initiated transcription, we challenged a tethered system encoding a 70 bp RNA with a different (unmodified) promoter-containing a DNA duplex that drives synthesis of a 34mer runoff RNA (with a sequence that allows only low levels of *cis*-primed extension), as shown in Figure 3A. Free enzyme should bind and transcribe from both the 70mer-encoding DNA and the 34mer-encoding challenge DNA, while tethered enzyme should transcribe only/primarily from the DNA to which it is tethered, producing the 70mer RNA.

The results shown in Figure 3B and quantified in Figure 3C confirm that as expected, at 0 M added salt, the untethered (free in solution) system shows both synthesis of both 70mer and 34mer RNA, and the presence of 0.4 M added salt significantly reduces synthesis from both. For the complex incubated to allow tethering via spontaneous crosslinking of the DNA-attached alkyl halide to the HaloTag domain, at 0 M added salt, competitive synthesis of the challenge 34mer RNA is reduced significantly. As above, production of the 70mer RNA remains largely resistant to a 0.4 M added salt challenge. To test whether residual 34mer RNA is produced from uncrosslinked species, this sample was washed (by magnetic bead pull down) before transcription to remove residual free polymerase. The crosslinked and washed complex now shows very little 34mer RNA, confirming that crosslinked RNA polymerase returns almost exclusively to its tethered RNA polymerase.

That crosslinked RNA polymerase strongly favors initiation from its tethered promoter predicts also that it will favor transcription of the desired product over the rebinding of free RNA to generate cis-primed (partially double stranded) extension products. The results in Figure 3B and C confirm this expectation in that even at no added salt, the tethered and washed complex shows a significant reduction in cis-prime extended products. Closer examination suggests, however, that even for the tethered and washed complex, some RNA is chased to extension products at 0 M added salt, as addition of salt nets an increase in the desired 70mer RNA. This observation is replicated in a repeat of the reaction, shown in Supplementary Figure S4.

We conclude from this that crosslinking the promoter DNA near its promoter binding site does indeed favor its rebinding over the rebinding of promoter DNA free in solution, and over the rebinding of accumulating product RNA. This sets the stage for new approaches to synthesis.

### Tethering allows repeat batch synthesis

The data above show that tethering promoter DNA near T7 RNA polymerase drives binding (via local proximity), allowing both increased resistance to salt and favoring initiation within the co-complex. Additionally, binding the co-tethered complex to magnetic beads via association of the DNA allows for the removal of untethered RNA polymerase, and also provides an additional important benefit: reuse of the complex for repeat batch synthesis. This is expected to allow production of large quantities of runoff RNA from relatively small amounts of DNA and enzyme, making the system more affordable compared to traditional, single round batch transcription.

**Figure 4.**
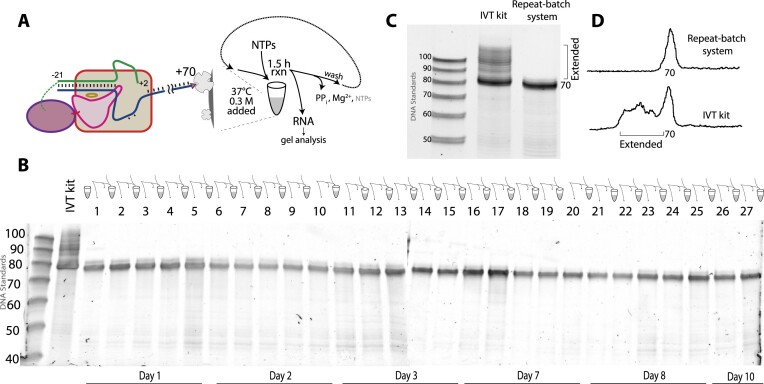
Bead tethered system can be reused multiple times without loss of activity. (**A**) Schematic diagram of repeat batch synthesis. (**B**) Transcription profile of repeat batch transcription of 70mer RNA over a span of 10 days. (**C**) Transcription profile of IVT kit and a pool of rounds 1–13 of repeat batch synthesis (equal total amounts of RNA loaded) after only desalting column purification. (**D**) The individual lane tracings of data in (C) quantify retention of transcription.

For synthesis of 70mer RNA, the approach is outlined in Figure 4A. Here we used the same biotin modified template (Figure 3) that encodes a 70mer RNA. After assembling and isolating the bead-bound binary complex, transcription buffer with 0.3 M added NaCl and containing NTPs, pyrophosphatase and RNase was added to initiate a first round of batch transcription. After the first synthesis, at 1.5 h, we removed the solution containing product RNA, phosphate, unreacted NTPs and buffer components. Addition of fresh reagents allows a second round of synthesis. The results from 27 rounds of synthesis are presented in Figure 4B and clearly demonstrate very efficient reuse of the complex over 10 days (with storage at 4°C between days), confirming exceptional stability of the immobilized complex.

While the initial yield from the bead-tethered system was reduced somewhat (presumably due to binding capacity limitations), the overall yield per DNA (and yield per enzyme) is dramatically higher than in the traditional approach. This system is necessarily 1:1 enzyme:DNA in active complexes, and so can show lower yields than in systems with excess enzyme, where multiple enzymes can transcribe from one DNA. Excess enzyme, however, presents the possibility that trailing enzymes might displace leading enzymes, leading to truncated products (33).

As predicted from earlier results, transcription in each round shows substantially less *cis-*primed extension products than in the free in solution (‘IVT kit’) control. In Figure 4C (with quantitative traces in Figure 4D) we compare RNA from the ‘IVT kit’ batch reaction, with RNA from a pooling of rounds 1–13 in the repeat batch reaction. As before, extended products are dramatically reduced. In addition, starting with the same amount of DNA, the IVT reaction yielded 128 μg RNA, while in contrast, the total amount of pooled RNA in the first 13 rounds of repeat batch synthesis is about 1200 μg RNA. To remove the extended products, the former would require extensive ‘lossy’ purification, while the latter requires no purification. This represents an approximately 10-fold increase in yield of RNA per DNA (and per enzyme).

To demonstrate the robustness and versatility of our approach, we have also generated a 104 bp guide RNA in a tethered system like that above. Transcription results, shown in Supplementary Figure S5, demonstrate good salt resistance out to 0.3–0.4 M NaCl, as in the experiments above.

### Extending the system to 1 kb mRNA

Once we confirmed the efficient synthesis of small RNAs and guide RNAs using the tethered HaloTag system, we proceeded to extend this novel approach to the synthesis of a longer (961 bp) mRNA encoding nanoluciferase enzyme (NLuc mRNA). To achieve this, we generated the promoter DNA by PCR (from an original plasmid DNA), using a forward (upstream) primer containing a 5′ chloroalkyl modification and a reverse (downstream) primer containing a 5′ biotin tag. The former effectively places a chloroalkyl group at the 5′ terminus (at position –21) of the nontemplate strand, while the latter allows coupling via the template strand to streptavidin-coated magnetic beads, as above.

**Figure 5.**
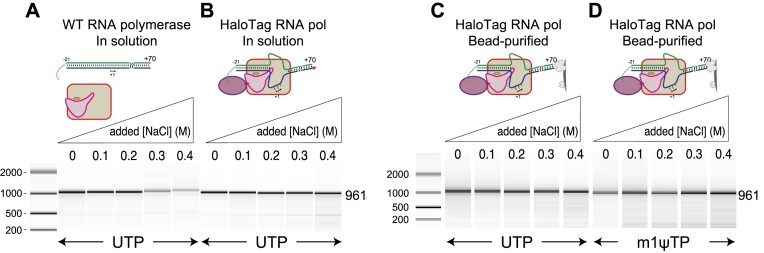
Extension to 1 kb mRNA encoding nanoluciferase. Salt challenge (added [NaCl] as noted) of transcription. (**A**) RNA polymerase (0.15 μM) and DNA (0.3 μM) free in solution. (**B**) HaloTag-coupled complex (not fully crosslinked). (**C**) As in (B) but functional complex purified by magnetic bead pull-down. (**D**) As in (C), but with N1-methyl-pseudo UTP replacing UTP in the reaction. In (C, D), starting concentrations, before coupling, were 0.5 μM polymerase and 0.15 μM template DNA (excess enzyme was washed away). Reactions were carried out for 2 h at 37°C in a reaction mix as described in Materials and methods.

As above, transcription with the crosslinked system and uncrosslinked control were challenged with increasing concentrations of added salt. As in Figure 2, transcription with the uncrosslinked control, shown in Figure 5A, displays salt sensitivity, while transcription with the crosslinked complex, shown in Figure 5B, shows significant salt resistance up to 0.4 M added NaCl. Again, the use of a magnetic bead pull down wash (in high salt buffer) removes any unbound RNA polymerase and should yield only the coupled system (and perhaps free DNA). The resulting purified complex shows good salt resistance, as shown in Figure 5C. Finally, to demonstrate applicability to functional mRNAs, the same functionally co-tethered system also performs well in transcription substituting UTP by N1-methylpseudo-UTP (m1ψ), as shown in Figure 5D.

### High salt, co-tethered transcription reduces RNA immunogenicity and increases cellular expression

As predicted by the high effective local concentrations (relative to each other) of the DNA and protein binding partners, the functionally crosslinked system above displays significant resistance to salt. As demonstrated with short RNA’s, co-tethering and added salt should reduce the RNA rebinding that leads to the generation of dsRNA. Since dsRNA triggers the innate immune response and that response down-regulates translation of mRNA in cells, we expect that increased purity of the mRNA will both decrease activation of receptors and increase expression of the NLuc gene (34,35).

**Figure 6.**
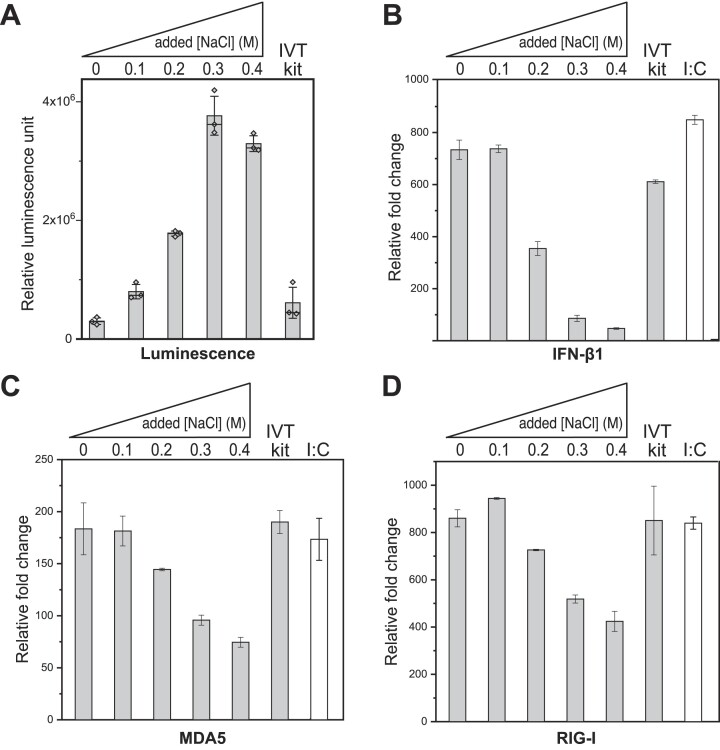
Nanoluciferase activity and immune response. RNA was transcribed under the added salt concentrations indicated and then samples were normalized to equivalent concentrations. Transcription with a standard New England Biolabs kit and poly(I:C) serves as controls. (**A**) Nanoluciferase activity of NLuc mRNAs transfected (100 ng RNA) into HEK293T cells. Luminescence was assayed 48 h post transfection. (**B**–**D**) RT-qPCR assays for expression of genes know to be upregulated by dsRNA. IFN-β1measures overall immune response. MDA5 and RIG-I are cytosolic receptors that respond to dsRNA. Relative fold change is measured using delta-delta C_t_ method (${{2}^{ - \Delta \Delta {{C}_t}}}$).

The RNAs in Figure 5 contain 5′ and 3′ untranslated regions and an encoded polyA tail. Samples from the reaction shown in Figure 5C were treated with the vaccina capping system (NEB) and mRNA cap 2′-*O*-methyltransferase (NEB) to enzymatically cap the 5′ end of the mRNA in each sample (producing a cap-1 structure). Finally, with no purification to remove dsRNA, we transfected HEK293T cells with equal amounts (100 ng) of each batch of NLuc mRNA and grew the cells at 37°C. After 48 h post transfection, we added luciferase assay substrate (Furimazine). The results in Figure 6A show the luciferase activity (relative luminescence) from the transfected cells at 48 h, as a function of added salt concentration in the transcription reaction. Consistent with the expectation that increasing salt decreases dsRNA production, and that dsRNA can repress translation in cells (36,37), expression of the mRNA increases with increasing salt in the transcription mix, up to about 0.3 M added NaCl. mRNA expressed at elevated salt also expresses significantly better (Supplementary Table S4) than mRNA prepared with a commonly used kit (all results are with no dsRNA purification, post-synthesis).

To assess the expected reduction in immunogenicity more directly, we next performed reverse transcriptase-mediated quantitative polymerase chain reaction (RT-qPCR) to measure the up-regulation of immune response indicators in HEK293T cells (38–43). We evaluated expression of type-I interferon beta (IFN-β1) and two cytosolic receptors involved in the innate immune response to RNA impurities: retionic acid-inducible gene I (RIG-I) and melanoma differentiation-associated protein 5 (MDA5). The charts in Figure 6 show the IFN-β1, MDA5 and RIG-I responses (panels B, C and D, respectively) in HEK293T cells transfected with NLuc mRNA originally synthesized at different NaCl concentrations. As expected, the response indicators decrease significantly (Supplementary Table S4) from 0.1 to 0.3 M added NaCl, consistent with a salt-dependent reduction in dsRNA at synthesis.

### Repeat batch synthesis of 1 kilobase mRNA using co-tethered complex

**Figure 7.**
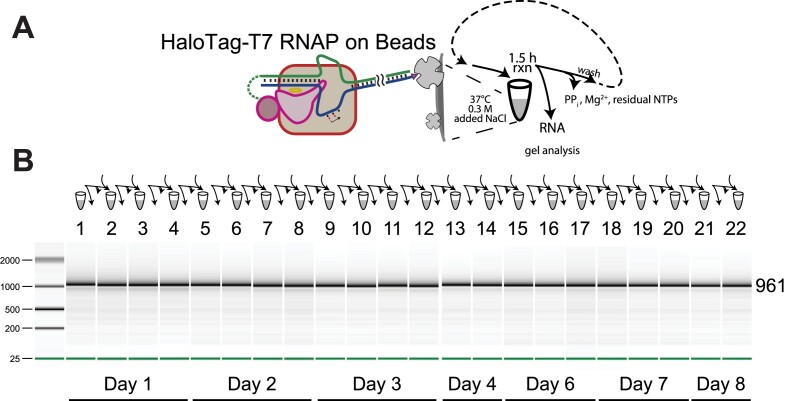
Repeat batch-IVT of NLuc mRNA using co-tethered complex. (**A**) Schematic representation of the co-tethered complex on a Streptavidin magnetic bead. (**B**) Transcription profile of repeat batch synthesis, analyzed with the 2100 Bioanalyzer system (Agilent).

To demonstrate the robustness of the approach, we next used the Streptavidin bead-bound, co-tethered complex to perform repeat batch *in vitro* transcription of NLuc mRNA as shown above for the 70-mer RNA (Figure 4B). The approach is presented in Figure 7A and we have performed 22 rounds of transcription using the same enzyme-promoter complex over a period of 8 days with the beads being stored at 4°C. The results show consistent transcription across batches.

## Discussion

mRNA therapeutics is emerging as a key next-generation biologics platform, owing in part to its unique potential for target-agnostic RNA generation, easy scalability, and broad applicability (44). RNA can and has been produced in small and large quantities using T7 RNA polymerase, a simple and fast single subunit enzyme (45,46). It has long been known, however, that in high yield batch reactions, the enzyme can rebind RNA and extend it, using RNA as the template (1,2,5). This results in regions of dsRNA of sufficient length to trigger the innate immune response, leading to inflammation and down-regulation of translation (3,9,34,35). Currently implemented efforts to reduce dsRNA center mostly on the use of modified bases (47) and on post-synthesis purification (11,12,48).

Tethering RNA polymerase to its promoter allows for transcription at salt concentrations that inhibit the RNA rebinding necessary for dsRNA synthesis. In our earliest tethering, we coupled T7 RNA polymerase to promoter DNA through a disulfide crosslink, but since the enzyme has 12 native cysteines, coupling was inefficient and likely heterogeneous (30). In more recent studies specifically targeting the dsRNA problem, we employed a Strep-Tag (short peptide) fusion with T7 RNA polymerase to tether biotin-tagged DNA and RNA polymerase to (adjacent sites on) Strep-Tactin®XT coated beads (21). The mutations introduced in the development of Strep-Tactin®XT, however, weaken the noncovalent biotin binding, limiting the approach in repeat-use (or flow) transcription.

In the current work, we instead employ a direct covalent coupling of T7 RNA polymerase to DNA by introducing a HaloTag domain fused to the N-terminus of the polymerase and coupling an alkyl-halide tether on the DNA to the HaloTag domain active site, as shown in Figure 1. With this covalent coupling, we can then tether the binary complex to a solid support using a (native) biotin-streptavidin interaction.

While there is no solved structure of the fusion protein, the rough modeling in Figure 1B is consistent with the observation that promoter-initiated transcription is efficient and exhibits higher salt tolerance than the uncoupled species, as revealed in Figure 2. That promoter binding is favored by local tethering is confirmed by the results shown in Figure 3, illustrating that RNA polymerase favors the duplex template to which it is bound over a template that is free in solution. Tethering the DNA (at the downstream end) to magnetic beads allows removal of traces of free RNA polymerase (compare, for example, competing transcription profiles at 0 M added NaCl for the unwashed vs washed species, shown in Figure 3C).

The intrinsic benefit of local tethering combined with the benefits of challenging RNA rebinding with added NaCl dramatically reduces the generation of dsRNA impurities, as shown in Figures 3B, C and 4C, D (challenged with (0.4 M and 0.3 M NaCl, respectively). The establishment of a system in which the co-tethered polymerase-promoter complex is bound to a solid support not only allows for removal of trace untethered RNA polymerase, but also allows repeat use of both enzyme and DNA in the synthesis of RNA, as illustrated in Figure 4B. In only 13 rounds of repeat synthesis, we obtain ≈20× higher overall yield (per enzyme and DNA) of the product RNA.

### Extension to long RNAs

In the above, we have demonstrated tethered synthesis of 34, 70 and (in Supplementary Figure S5) 104 bp RNA. In these experiments, the alkyl-halide and biotin ‘handles’ were included directly in the oligonucleotide nontemplate and template strands prior to assembly of the functional (partial) duplexes. To demonstrate the synthesis of mRNA lengths, we created a plasmid DNA template encoding a 961 base mRNA, which encodes the nanoluciferase protein. The ‘handles’ were incorporated into PCR primers that drive amplification of the DNA. Salt challenge results paralleling experiments above are shown in Figure 5A-C and show the same increased trend in salt tolerance. We also show, in Figure 5D, synthesis of mRNA in which N1-methyl-pseudouridine replaces U.

While gel electrophoresis allows resolution of (at least some) RNA extensions from RNAs up to at least 70 bases in length, resolution of 1 kb RNA extensions is more problematic, in part due to the longer lengths and likely in part since 3′ extensions are expected to be (even more) polydisperse in length. To assess the reduction in dsRNA extended products, we instead turned to assays in mammalian cell culture. dsRNA regions longer than about 40 base pairs activate the innate immune response, repressing translation and upregulating a variety of response genes. Paralleling the gel results for short RNAs, increasing salt (in a co-tethered system) results in increased translation (activity) of the nanoluciferase enzyme and decreased upregulation of the innate immune response reporters, for equal concentrations of RNA transfected, as shown in Figure 6. The cell-based assays support the biochemical results, but more importantly, point the way towards the synthesis (manufacturing) of vaccine and therapeutic mRNAs. Finally, it is noteworthy that the cell-based assays were conducted using mRNA transcribed without modified bases and without significant purification post mRNA synthesis.

Paralleling the repeat-use results presented in Figure 4 for 70 bp RNA, a similar experiment with 1 kb mRNA presented in Figure 7 shows promising utility in bringing down the cost of mRNA manufacturing. As before, a given amount of enzyme and DNA can generate much more RNA than in a traditional, single use reaction. The fact that activity remains approximately constant over at least 22 synthesis/wash cycles also indicates that very little enzyme and DNA is likely to be contaminating the product mRNA (consistent with the relatively low immunogenicity of RNAs generated at high salt). As in the prior experiments, post-synthesis purification was limited to simple desalting.

## Summary

These findings affirm the following key advantages of covalently tethering RNA polymerase to promoter DNA and tethering the co-complex to a surface: the approach (a) offers a straightforward and efficient method for creating RNA of any size and sequence; (b) enables the production of mRNA incorporating modified nucleobases (if desired), (c) requires significantly less DNA and RNA polymerase (with less contamination in the output), (d) offers a greatly simplified overall workflow, eliminating purification steps to remove RNA polymerase and DNA and (e) achieves the production of high-quality mRNA with minimal to negligible dsRNA impurities. While this approach currently requires generation of the reagent described in Figure S1, this universal reagent can be used across many different RNA syntheses. Finally, this system could be used to generate an efficient continuous flow reactor for the synthesis of siRNA, CRISPR guide RNA, long noncoding RNAs, and of course, mRNA for vaccines and therapeutics.

## Supplementary Material

gkae599_Supplemental_File

## Data Availability

There are no new data associated with this article.
