# Supplementary material for: A new approach to RNA synthesis: immobilization of stably and functionally co-tethered promoter DNA and T7 RNA polymerase
Source: Nucleic Acids Res. 2024 Jul 16;52(17):10607–18. doi: 10.1093/nar/gkae599 (PMC11417385; doi:10.1093/nar/gkae599)
Supplement: gkae599_Supplemental_File [file gkae599_supplemental_file.docx]

**Supplementary**

**Supplementary Table I: Sequences of DNA used in Figures 2, 3, and 4**

| **Name** | **Description** | **Sequence (5’→ 3’)** |
| --- | --- | --- |
| 34-NT  70-NT  104-NT | Nontemplate Strand (paired with 34-T, 70T, or 104-T) | 5’ Amino Modifier C6-AATTAATACGACTCACTATAGG |
| 34-T | 34mer Template Strand | 5’ Biotin TEG-TAAATGCGTCGACGTAGTTTCGAGTCATACCTCCTATAGTGAGTCGTATTAATT |
| 70-T | 70mer Template Strand | 5’ Biotin TEG-TAAATCTTCACCTCTACTACCTCCAAGCACGAACTTGCTATTCTAGCTTACAGTCGTATTAATTTCCTCCTATAGTGAGTCGTATTAATT |
| 104-T | 104mer Template Strand | 5’ Biotin-Int Spacer 18-AAAAGCACCGACTCGGTGCCACTTTTTCAAGTTGATAACGGACTAGCCTTATTTTAACTGCTATTTCTAGCTCTAAAACATAGTGAGTCGTATTAATTTCCTCCTATATGAGTCGTATTAATT |

All sequences are written in the 5’ to 3’ direction. Nomenclature of modifications follows product names at IDT. Note that in keeping with common practice, a single nontemplate (NT) strand was paired with three different template strands (34-T, 70-T, and 104-T) to generate partially duplex constructs for transcription.

**Supplementary Table II: DNA primer sequences used in Figures 5, 6 and 7.**

1. Original DNA sequence for nanoluciferase-encoding (NLuc) mRNA. DNA sequence (nontemplate, contains a TEV cleavage site and a 6x -His Tag) from plasmid NanoLuc-SZ2 (Addgene plasmid #176645)

5’ATGGTCTTCACACTCGAAGATTTCGTTGGGGACTGGCGACAGACAGCCGGCTACAACCTGGACCAAGTCCTTGAACAGGGAGGTGTGTCCAGTTTGTTTCAGAATCTCGGGGTGTCCGTAACTCCGATCCAAAGGATTGTCCTGAGCGGTGAAAATGGGCTGAAGATCGACATCCATGTCATCATCCCGTATGAAGGTCTGAGCGGCGACCAAATGGGCCAGATCGAAAAAATTTTTAAGGTGGTGTACCCTGTGGATGATCATCACTTTAAGGTGATCCTGCACTATGGCACACTGGTAATCGACGGGGTTACGCCGAACATGATCGACTATTTCGGACGGCCGTATGAAGGCATCGCCGTGTTCGACGGCAAAAAGATCACTGTAACAGGGACCCTGTGGAACGGCAACAAAATTATCGACGAGCGCCTGATCAACCCCGACGGCTCCCTGCTGTTCCGAGTAACCATCAACGGAGTGACCGGCTGGCGGCTGTGCGAACGCATTCTGGCGGGCGGCGGATCTGAGAATTTATATTTCCAGGGCGCTCGTAATGCATACCTGCGTAAAAAAATCGCGCGTTTAAAAAAAGACAACTTGCAACTTGAGCGCGACGAACAAAATTTGGAGAAAATAATCGCCAACCTTCGGGACGAGATCGCGCGTCTGGAAAACGAGGTGGCTTCGCATGAGCAACTCGAGCACCACCACCACCACCACTGA-3’

1. Engineered DNA construct including the above DNA sequence encoding NLuc mRNA following a consensus **T7 RNA polymerase promoter**, 5’ UTR, and preceding a 3’ UTR and an encoded poly-A tail. The encoded mRNA begins with 5’-AG, allowing (requiring) the use of CleanCap® Reagent AG as an initiator.

5’GTGCGCGGTAAT**TAATACGACTCACTATA**AGAAATAAGAGAGAAAAGAAGAGTAAGAAGAAATATAAGAGCCACCATGGTCTTCACACTCGAAGATTTCGTTGGGGACTGGCGACAGACAGCCGGCTACAACCTGGACCAAGTCCTTGAACAGGGAGGTGTGTCCAGTTTGTTTCAGAATCTCGGGGTGTCCGTAACTCCGATCCAAAGGATTGTCCTGAGCGGTGAAAATGGGCTGAAGATCGACATCCATGTCATCATCCCGTATGAAGGTCTGAGCGGCGACCAAATGGGCCAGATCGAAAAAATTTTTAAGGTGGTGTACCCTGTGGATGATCATCACTTTAAGGTGATCCTGCACTATGGCACACTGGTAATCGACGGGGTTACGCCGAACATGATCGACTATTTCGGACGGCCGTATGAAGGCATCGCCGTGTTCGACGGCAAAAAGATCACTGTAACAGGGACCCTGTGGAACGGCAACAAAATTATCGACGAGCGCCTGATCAACCCCGACGGCTCCCTGCTGTTCCGAGTAACCATCAACGGAGTGACCGGCTGGCGGCTGTGCGAACGCATTCTGGCGGGCGGCGGATCTGAGAATTTATATTTCCAGGGCGCTCGTAATGCATACCTGCGTAAAAAAATCGCGCGTTTAAAAAAAGACAACTTGCAACTTGAGCGCGACGAACAAAATTTGGAGAAAATAATCGCCAACCTTCGGGACGAGATCGCGCGTCTGGAAAACGAGGTGGCTTCGCATGAGCAACTCGAGCACCACCACCACCACCACTGAGCTCGCTTTCTTGCTGTCCAATTTCTATTAAAGGTTCCTTTGTTCCCTAAGTCCAACTACTAAACTGGGGATATTATGAAGGGCCTTGAGCATCTGGATTCTGCCTAATAAAAAACATTTATTTTCATTGCAAAAAAAAAAAAAAAAAAAAAAAAAAAAAAAAAAAAAAAAAAAAAAAAAAAAAAAAAAAA-3’

1. Primers used to PCR amplify the above DNA and allowing the incorporation of “handles” into the resultant DNA.

| **Name** | **Description** | **Sequence (5’→ 3’)** |
| --- | --- | --- |
| NLuc FP | NLuc Forward Primer | GTGCGCGGTAATTAATACGACTCACTATAAGAAATAAGAGAGAAAAGAAGAGTAAGAAGAAATATAAGAGCCACC |
| NLuc FP NH2 | NLuc Forward Primer Amine | 5’ Amino Modifier C12-(Int Spacer 18)_4_-GTGCGCGGTAATTAATACGACTCACTATAAGAAATAAGAGAGAAAAGAAGAGTAAGAAGAAATATAAGAGCCACC |
| NLuc RP 1 | NLuc Reverse Primer 1 | AGTAGTTGGACTTAGGGAACAAAGGAACCTTTAATAGAAATTGGACAGCAAGAAAGCGAGCTCAGTGGTGGTGGTGGTGGTGCTCGAGTT |
| NLuc RP 2 | NLuc Reverse Primer 2 | AGATGCTCAAGGCCCTTCATAATATCCCCAGTTTAGTAGTTGGACTTAGGGAACAAAGG |
| NLuc RP 3 | NLuc Reverse Primer 3 | GCAATGAAAATAAATGTTTTTTATTAGGCAGAATCCAGATGCTCAAGGCCCTTC |
| NLuc RP 4 | NLuc Reverse Primer 4 | TTTTTTTTTTTTTTTTTTTTTTTTTTTTTTTTTTTTTTTTTTTTTTTTTTTTTTTTTTTTGCAATGAAAATAAATGTTTTTTATTAGGCA |
| NLuc Bio-RP 4 | NLuc Reverse Primer 4-biotin | 5’ Biotin-Int Spacer 18-TTTTTTTTTTTTTTTTTTTTTTTTTTTTTTTTTTTTTTTTTTTTTTTTTTTTTTTTTTTTGCAATGAAAATAAATGTTTTTTATTAGGCA |

All sequences are written in the 5’ to 3’ direction. Nomenclature of modifications follows product names at IDT.

**Supplementary Table III: DNA primer sequences used in RT-qPCR (Figure 6 B,C & D)**

| **Gene** | **Forward/Reverse** | **Sequence (5’→ 3’)** |
| --- | --- | --- |
| *GAPDH* | Forward | ATTCCACCCATGGCAAATTC |
|  | Reverse | TGGGATTTCCATTGATGACAAG |
| *IFNB1* | Forward | ttcagtgtcagaagctcctgtgg |
|  | Reverse | ctgcttaatctcctcagggatgtca |
| *MDA5 (IFIH1)* | Forward | aggaggaactgttgacaattg |
|  | Reverse | agtagctctcttacacctgattc |
| *RIG-I (DDX58)* | Forward | tggaccctacctacatcctg |
|  | Reverse | tcagcctgaatatactgcac |

**Supplementary Table IV: Statistical analysis of Figure-6**

Tukey’s Honest Significant Difference (HSD) is used to quantify p-value which is tabulated below. Significance levels: *** (p<0.0005), ** (p<0.005), * (p<0.05), n.s. (not significant)

| \| **Nanoluciferase expression** \| \| \| \| --- \| --- \| --- \| \| Group-1 \| Group-2 \| p-value \| \| 0 mM \| 100 mM \| n.s. \| \| 200 mM \| *** \| \| 300 mM \| *** \| \| 400 mM \| *** \| \| IVT Kit \| *** \| \| 100 mM \| 200 mM \| ** \| \| 300 mM \| *** \| \| 400 mM \| *** \| \| IVT Kit \| n.s. \| \| 200 mM \| 300 mM \| *** \| \| 400 mM \| *** \| \| IVT Kit \| *** \| \| 300 mM \| 400 mM \| n.s. \| \| IVT Kit \| *** \| \| 400 mM \| IVT Kit \| *** \| | \| **IFN-β1** \| \| \| \| --- \| --- \| --- \| \| Group-1 \| Group-2 \| p-value \| \| 0 mM \| 100 mM \| n.s. \| \| 200 mM \| *** \| \| 300 mM \| *** \| \| 400 mM \| *** \| \| IVT Kit \| ** \| \| Poly I:C \| ** \| \| 100 mM \| 200 mM \| *** \| \| 300 mM \| *** \| \| 400 mM \| *** \| \| IVT Kit \| ** \| \| Poly I:C \| ** \| \| 200 mM \| 300 mM \| *** \| \| 400 mM \| *** \| \| IVT Kit \| *** \| \| Poly I:C \| *** \| \| 300 mM \| 400 mM \| n.s. \| \| IVT Kit \| *** \| \| Poly I:C \| *** \| \| 400 mM \| IVT Kit \| *** \| \| Poly I:C \| *** \| \| IVT Kit \| Poly I:C \| *** \| |
| --- | --- | --- | --- | --- | --- | --- | --- | --- | --- | --- | --- | --- | --- | --- | --- | --- | --- | --- | --- | --- | --- | --- | --- | --- | --- | --- | --- | --- | --- | --- | --- | --- | --- | --- | --- | --- | --- | --- | --- | --- | --- | --- | --- | --- | --- | --- | --- | --- | --- | --- | --- | --- | --- | --- | --- | --- | --- | --- | --- | --- | --- | --- | --- | --- | --- | --- | --- | --- | --- | --- | --- | --- | --- | --- | --- | --- | --- | --- | --- | --- | --- | --- | --- | --- | --- | --- | --- | --- | --- | --- | --- | --- | --- | --- | --- | --- |

| \| **MDA5** \| \| \| \| --- \| --- \| --- \| \| Group-1 \| Group-2 \| p-value \| \| 0 mM \| 100 mM \| n.s. \| \| 200 mM \| n.s. \| \| 300 mM \| ** \| \| 400 mM \| ** \| \| IVT Kit \| n.s. \| \| Poly I:C \| n.s. \| \| 100 mM \| 200 mM \| n.s. \| \| 300 mM \| ** \| \| 400 mM \| ** \| \| IVT Kit \| n.s. \| \| Poly I:C \| n.s. \| \| 200 mM \| 300 mM \| n.s. \| \| 400 mM \| * \| \| IVT Kit \| n.s. \| \| Poly I:C \| n.s. \| \| 300 mM \| 400 mM \| n.s. \| \| IVT Kit \| ** \| \| Poly I:C \| ** \| \| 400 mM \| IVT Kit \| *** \| \| Poly I:C \| ** \| \| IVT Kit \| Poly I:C \| n.s. \| | \| **RIG-I** \| \| \| \| --- \| --- \| --- \| \| Group-1 \| Group-2 \| p-value \| \| 0 mM \| 100 mM \| n.s. \| \| 200 mM \| n.s. \| \| 300 mM \| ** \| \| 400 mM \| ** \| \| IVT Kit \| n.s. \| \| Poly I:C \| n.s. \| \| 100 mM \| 200 mM \| n.s. \| \| 300 mM \| ** \| \| 400 mM \| *** \| \| IVT Kit \| n.s. \| \| Poly I:C \| n.s. \| \| 200 mM \| 300 mM \| n.s. \| \| 400 mM \| * \| \| IVT Kit \| n.s. \| \| Poly I:C \| n.s. \| \| 300 mM \| 400 mM \| n.s. \| \| IVT Kit \| ** \| \| Poly I:C \| * \| \| 400 mM \| IVT Kit \| ** \| \| Poly I:C \| ** \| \| IVT Kit \| Poly I:C \| n.s. \| |
| --- | --- | --- | --- | --- | --- | --- | --- | --- | --- | --- | --- | --- | --- | --- | --- | --- | --- | --- | --- | --- | --- | --- | --- | --- | --- | --- | --- | --- | --- | --- | --- | --- | --- | --- | --- | --- | --- | --- | --- | --- | --- | --- | --- | --- | --- | --- | --- | --- | --- | --- | --- | --- | --- | --- | --- | --- | --- | --- | --- | --- | --- | --- | --- | --- | --- | --- | --- | --- | --- | --- | --- | --- | --- | --- | --- | --- | --- | --- | --- | --- | --- | --- | --- | --- | --- | --- | --- | --- | --- | --- | --- | --- | --- | --- | --- | --- | --- | --- | --- | --- | --- | --- | --- | --- | --- | --- | --- | --- | --- |

**Supplementary Figure S1**. **Addition of the halo-alkane linkage to 5’-amino-modified oligonucleotide DNA**. The top and bottom pairs illustrate formation of the Cl-C_12_ and Cl-C_12_-(Spacer 18)_4_ adducts, respectively.

**Supplementary Figure S2. Confirmation of transcription with promoter DNA and RNA polymerase adducts**. A) The DNA construct used is fully double stranded only to position +2 and encodes a 34mer RNA. B) Synthesis of 34mer RNA from assembled DNA with or without an alkyl-Cl at the 5’ position of the nontemplate DNA, using native T7 RNA polymerase or its HaloTag-fused variant. Transcripts were labeled with [α-^32^P]ATP and were resolved on a 7M urea, 20% acrylamide sequencing gel.

**Supplementary Figure S3. Characterization of HaloTag-T7 RNA polymerase assembly on beads**. A) 10% SDS protein gel shows that the band representing T7 RNA polymerase shifts fully on binding the alkyl halide-containing nontemplate DNA (Before Wash), indicating efficient coupling. Assembly with the template strand bound to beads allows washing. Flow through (Unbound) and repeat washes (1-3) show little free RNA polymerase. Assay of polymerase on beads (“Bound) confirms that protein is bound to the beads. DNase treatment releases most of the protein (Solution After DNase Treatment compared with Bead after DNase treatment). B) As a control, a 10% SDS protein gel shows that native T7 RNA polymerase (lacking a HaloTag fusion) assembled as above fails to bind beads.


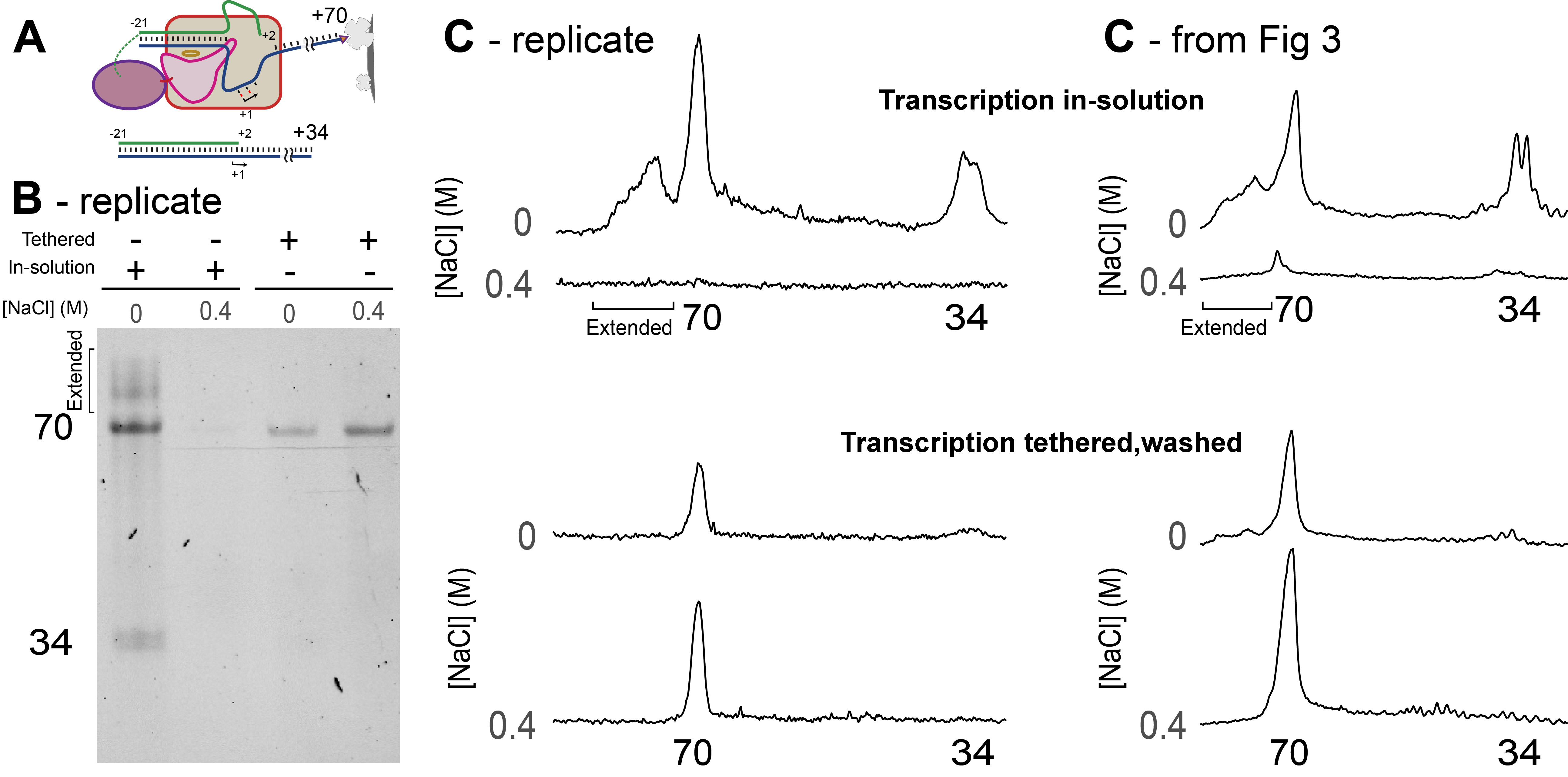


**Supplementary Figure S4. Replicate of Figure 3 – tethering drives transcription to crosslinked promoter DNA.** Panels A-C are as in Figure 3, except that tethered, but not washed is omitted. Data at right in (C) are reproduced from Figure 3 for ease of comparison with replicate traces.

**Supplementary Figure S5. Transcription of 104 base guide RNA.** Salt resistance of transcription with a fully tethered (washed to remove polymerase that is not covalently bound) system driving synthesis of a 104 base guide RNA.
